# Mortality and length of stay associated with antimicrobial-susceptible and -resistant hospital-onset bloodstream infections at a tertiary referral hospital in Melbourne, Australia

**DOI:** 10.1093/jacamr/dlaf183

**Published:** 2025-10-21

**Authors:** Stephanie J Curtis, Sue J Lee, Ben S Cooper, Jan M Bell, Geoffrey W Coombs, Denise A Daley, Allen C Cheng, Denis W Spelman, Anton Y Peleg, Andrew J Stewardson

**Affiliations:** Department of Infectious Diseases, The Alfred Hospital and Monash University School of Translational Medicine, 55 Commercial Road, Melbourne, Victoria 3004, Australia; Department of Infectious Diseases, The Alfred Hospital and Monash University School of Translational Medicine, 55 Commercial Road, Melbourne, Victoria 3004, Australia; Centre for Tropical Medicine and Global Health, Nuffield Department of Medicine, University of Oxford— Old Road Campus, Roosevelt Drive, Oxford OX3 7LG, UK; Mahidol-Oxford Tropical Medicine Research Unit, Faculty of Tropical Medicine, Mahidol University, 10400 Bangkok, Thailand; Centre for Tropical Medicine and Global Health, Nuffield Department of Medicine, University of Oxford— Old Road Campus, Roosevelt Drive, Oxford OX3 7LG, UK; Mahidol-Oxford Tropical Medicine Research Unit, Faculty of Tropical Medicine, Mahidol University, 10400 Bangkok, Thailand; Australian Group on Antimicrobial Resistance, Canberra, ACT, Australia; Australian Group on Antimicrobial Resistance—Fiona Stanley Hospital, 9 Warren Robin Drive, Murdoch, Western Australia 6150, Australia; Department of Microbiology, PathWest Laboratory Medicine-WA—Fiona Stanley Hospital, 9 Warren Robin Drive, Murdoch, Western Australia 6150, Australia; School of Medical, Molecular and Forensic Sciences, Murdoch University, 90 South Street, Murdoch, Western Australia 6150, Australia; Australian Group on Antimicrobial Resistance—Fiona Stanley Hospital, 9 Warren Robin Drive, Murdoch, Western Australia 6150, Australia; Department of Microbiology, PathWest Laboratory Medicine-WA—Fiona Stanley Hospital, 9 Warren Robin Drive, Murdoch, Western Australia 6150, Australia; Infectious Diseases, Monash Health, 246 Clayton Road, Clayton, Victoria 3168, Australia; School of Clinical Sciences, Faculty of Medicine, Nursing and Health Sciences—Monash University, Clayton, Victoria 3800, Australia; Centre to Impact AMR, Monash University, Clayton, Victoria 3800, Australia; Department of Infectious Diseases, The Alfred Hospital and Monash University School of Translational Medicine, 55 Commercial Road, Melbourne, Victoria 3004, Australia; Microbiology Unit, Alfred Pathology Service, The Alfred, 55 Commercial Rd, Melbourne, Victoria 3004, Australia; Department of Infectious Diseases, The Alfred Hospital and Monash University School of Translational Medicine, 55 Commercial Road, Melbourne, Victoria 3004, Australia; Centre to Impact AMR, Monash University, Clayton, Victoria 3800, Australia; Department of Microbiology, Infection Program, Monash Biomedicine Discovery Institute, Monash University, Clayton, Victoria 3800, Australia; Department of Infectious Diseases, The Alfred Hospital and Monash University School of Translational Medicine, 55 Commercial Road, Melbourne, Victoria 3004, Australia; Centre to Impact AMR, Monash University, Clayton, Victoria 3800, Australia

## Abstract

**Background and objectives:**

There are few Australian data regarding the burden of hospital-onset bloodstream infections (HO-BSIs). To quantify the impact of antimicrobial-susceptible and -resistant HO-BSIs on patient outcomes by augmenting laboratory-based surveillance data.

**Methods:**

We performed a retrospective cohort study at a tertiary referral hospital in Melbourne, Australia, from 2015 to 2020. We linked administrative data with bloodstream infection surveillance data from the Australian Group on Antimicrobial Resistance. We performed cause-specific Cox proportional hazards regression to quantify the impact of HO-BSI on inpatient mortality and discharge alive, with separate models for Enterobacterales, *Staphylococcus aureus*, *Enterococcus* species and the non-fermenting Gram-negative bacilli (NFGNB), *Pseudomonas aeruginosa* and *Acinetobacter* species, compared to admissions without HO-BSI. Excess length of stay (LOS) was estimated using multistate models.

**Results:**

The cohort of 278 984 admissions included 814 (0.3%) HO-BSIs. Enterobacterales were the most frequent pathogens, followed by enterococci, *S. aureus* and NFGNB (incidence 3.62, 2.34, 1.11 and 0.80 events per 10 000 patient-days, respectively). Both antimicrobial-resistant and -susceptible HO-BSI increased risk of death and LOS compared with admissions without HO-BSI. Antimicrobial-resistant and -susceptible HO-BSIs, respectively, increased LOS by 5.7 days (95% CI: 4.9–6.5) and 4.1 days (95% CI: 3.8–4.5) for Enterobacterales, 4.9 days (95% CI: 4.5–5.4) and 3.1 days (95% CI: 2.6–3.6) for enterococci, and 6.3 days (95% CI: 5.3–7.3) and 9.8 days (95% CI: 9.1–10.5) for *S. aureus*.

**Conclusions:**

Antimicrobial-susceptible and -resistant HO-BSIs have a substantial impact on patient outcomes. We demonstrated the feasibility of leveraging a national laboratory-based surveillance system to quantify the impact of HO-BSI.

## Introduction

Healthcare-associated infections (HAIs) represent a substantial, and potentially preventable, threat to patient safety, with bloodstream infections (BSIs) resulting in the highest mortality among HAIs.^1^ This burden is compounded by the continued increase of antimicrobial resistance among HAIs.^2,3^ In Australia, it is mandatory for public hospitals to report the incidence of healthcare-associated *Staphylococcus aureus* BSI prospectively, but outcomes are not reported and healthcare-associated BSIs caused by other pathogens are not included. The Australian Group on Antimicrobial Resistance (AGAR) conduct laboratory-based surveillance of antimicrobial susceptibility among all BSIs caused by selected priority pathogens in Australian hospitals.^4^ Limited clinical data are collected through this surveillance system, including all-cause mortality.

Globally, there is scarcity of data regarding the relative impact of healthcare-associated BSIs caused by antimicrobial-susceptible and -resistant isolates on patient outcomes.^5^ There is also considerable heterogeneity between studies and a scarcity of research that uses robust epidemiological methods to appropriately adjust for time-dependent bias and account for competing risks.^5–7^ Existing estimates of the burden of BSIs stratified by antimicrobial susceptibility are focused on *S. aureus* and Enterobacterales and in North American regions.^8^ The situation in Australia cannot necessarily be extrapolated from international studies given differences in local epidemiology.^9,10^ Therefore, we aimed to estimate the burden of hospital-onset BSI (HO-BSI) caused by priority bacterial groups, stratified by antimicrobial susceptibility, at an Australian hospital by using data linkage to augment prospective AGAR surveillance.

## Methods

### Study design, setting and population

We performed a single-site retrospective cohort study of inpatient acute-care episodes at the Alfred Hospital in Melbourne, Australia, from 1 January 2015 to 31 December 2020. The Alfred is a major tertiary and quaternary referral hospital with the largest ICU in Australia. Quaternary services include heart and lung transplantation, and care for patients with cystic fibrosis, burns, HIV infection and major trauma. Ambulatory, hospital-in-the-home and non-acute care episodes, and emergency consultations without consequent hospital admission were excluded. Elective acute-care episodes that started and ended on the same calendar day were also excluded. Patients under 16 years of age were excluded as the Alfred Hospital does not usually provide inpatient care for children under this age.

### Data sources

We linked BSI surveillance data from the AGAR (for exposure data) with hospital administrative data (for covariate and outcome data). AGAR conducts ongoing surveillance of antibiotic susceptibility of priority pathogens isolated from blood cultures collected from patients either presenting to, or already admitted to, participating hospitals through three Sepsis Outcome Programs (SOPs): Gram-negative Surveillance Outcome Program (GnSOP), Australian Enterococcus Surveillance Outcome Program (AESOP) and *S. aureus* Surveillance Outcome Program (ASSOP).^4^ GnSOP includes Enterobacterales, *Pseudomonas aeruginosa* and *Acinetobacter* species. AESOP includes all *Enterococcus* species. The Alfred Hospital is 1 of 33 member laboratories across Australia that provide BSI data for the three SOPs. For this study, we extracted laboratory number, bacterial species, antimicrobial susceptibility testing and limited clinical information from the Alfred Health AGAR dataset.

Administrative data are routinely collected for all patient episodes at the Alfred Hospital. We extracted a dataset with covariates and the primary outcomes: in-hospital mortality and length of stay (LOS) in hospital. Routinely collected data also include the following covariates used in our analysis: age, sex, admission type, admission provenance, most recent previous separation date from Alfred Hospital, admission to intensive care, surgical procedure and comorbidities identified by International Classification of Diseases, Tenth Revision, Australian Modification (ICD-10-AM) codes.

### Data linkage

Both datasets included the following patient identifiers: unit record number (URN), date of birth and sex. In the case of AGAR data, URN was derived from the BSI laboratory number. Data were linked by matching two or more patient identifiers (URN, age, sex) and dates; a blood culture episode (from AGAR) was linked to a specific admitted episode (from hospital administrative data) if the two records involved the same patient, and the date of blood culture collection fell within the first and last calendar days (inclusive) of that admitted episode. In cases where the blood culture collection did not occur during an admitted episode, we linked the blood culture to the next admitted episode for that same patient if that admission commenced in the next two calendar days after blood culture collection. In cases where the patient was discharged and then admitted on the date of blood culture collection, we linked the blood culture to the second admitted episode, i.e. the episode that started on the day of blood culture collection. Where the laboratory data could not be linked to an administrative record through two or more matching patient identifiers, we performed a manual medical record review of records with at least one matching patient identifier to confirm matching records and used the patient identifiers in hospital administrative data where there were discrepancies between the datasets.

### Microbiological methods

Blood cultures were incubated using the BACT/ALERT system (bioMérieux, Marcy-Étoile, France). Positive cultures were plated onto a selection of agar plates according to their Gram stain result (including horse blood, chocolate, MacConkey and Sabouraud dextrose agar). Identification of isolated colonies was performed using MALDI-TOF MS (bioMérieux, Marcy-Étoile, France) and antimicrobial susceptibility testing performed using VITEK-2 AST cards (bioMérieux, version 8.01) or graduated antimicrobial test strips (E-tests, bioMérieux, Marcy-Étoile, France) interpreted using European Committee on Antimicrobial Susceptibility Testing (EUCAST).^11^

### Definitions

Once blood cultures were each linked to one patient admission episode, we defined them as hospital-onset if collected after the third calendar day of the admission or if the patient was transferred from a non-acute ward, facility or another hospital and the date of blood collection was on the first calendar day onwards following the transfer. All other BSIs were considered community-onset and were excluded from this analysis. BSI was defined as per the AGAR definition: the growth of the relevant bacteria in one or more blood cultures.^12^ In patients with more than one isolate of the same pathogen, AGAR defines a new episode as a new positive blood culture more than 2 weeks after the initial positive culture. Only the first positive blood culture for each new episode is included in the AGAR dataset. Key antibiotics to infer isolate resistance were third-generation cephalosporins for Enterobacterales, vancomycin for enterococci, and methicillin for *S. aureus*. Other non-fermenting Gram-negative bacilli (NFGNB) were not analysed by antimicrobial susceptibility due to small sample size.

### Statistical analysis

We used descriptive statistics to summarize covariates and estimate the incidence of HO-BSI caused by the following bacterial groups: Enterobacterales, enterococci, *S.aureus* and NFGNB (*Pseudomonas aeruginosa* and *Acinetobacter* species). We performed cause-specific Cox proportional hazards regression models to quantify the impact of HO-BSI on the competing events, in-hospital mortality and discharge alive. The exposure of interest, HO-BSI, was included as a time-varying covariate. We first performed the analysis for each bacterial group, regardless of antimicrobial susceptibility, and subsequently performed the analysis for antimicrobial-susceptible and antimicrobial-resistant isolates separately for Enterobacterales, enterococci and *S. aureus*. We aimed to quantify the impact of HO-BSIs, including susceptible and resistant HO-BSIs, compared with no infection. We did not directly estimate the impact of susceptible versus resistant infections among patients with HO-BSI. Where an admission had more than one HO-BSI, the index BSI was the first BSI specific to that model (i.e. for admissions with both a resistant and susceptible BSI, the resistant BSI was used in the resistant model and the susceptible BSI in the susceptible model) and the earlier of the two BSIs was use in the overall model.

In addition, we repeated the cause-specific models with adjustment for age (continuous), sex (male versus other), 17 Charlson comorbidity index conditions as dichotomous variables, admission source (home, from non-acute ward or facility, or from acute care), elective or emergency admission, discharge within the previous 30 days (yes/no), and two time-varying covariates: ICU admission or surgery while at risk for HO-BSI.^13^

As the unit of the analysis was hospital admission, we accounted for non-independence between admissions for the same patient by using the robust cluster variance (Huber–White sandwich) estimators. Hospital stays were censored at 45 days to reduce the influence of outliers.^4^ The proportional hazards assumption was assessed using Schoenfeld residuals and visually using log-log plots. Violations of the proportional hazards assumptions were managed using stratification. The results of all Cox models were expressed as unadjusted and adjusted HRs with 95% CIs.

We used multistate models with three separate states (admission, antimicrobial-resistant or -susceptible, and died/discharged) and three transitions to estimate the expected LOS in days.^14^ The expected LOS for each of the HO-BSI groups was computed for each day in each state in the antimicrobial-resistant or -susceptible and admitted (non-infected patients) states using Aalen–Johansen estimators for the transition probabilities. The difference in LOS was calculated between antimicrobial-resistant BSIs and non-infected patients, and between antimicrobial-susceptible BSIs and non-infected patients. The overall change in LOS was computed as a weighted average (mean) using the observed distribution of time to HO-BSI onset. Standard errors and 95% CIs were derived by bootstrap resampling runs. Statistical analyses were done using R version 4.3.0 (www.r-project.org/) and Stata 16.0 (https://www.stata.com) using the multistate package.^15^

### Ethics

The project was approved by the Alfred Health Human Research Ethics Committee (39/19).

## Results

### Description of cohort

**Figure 1.**
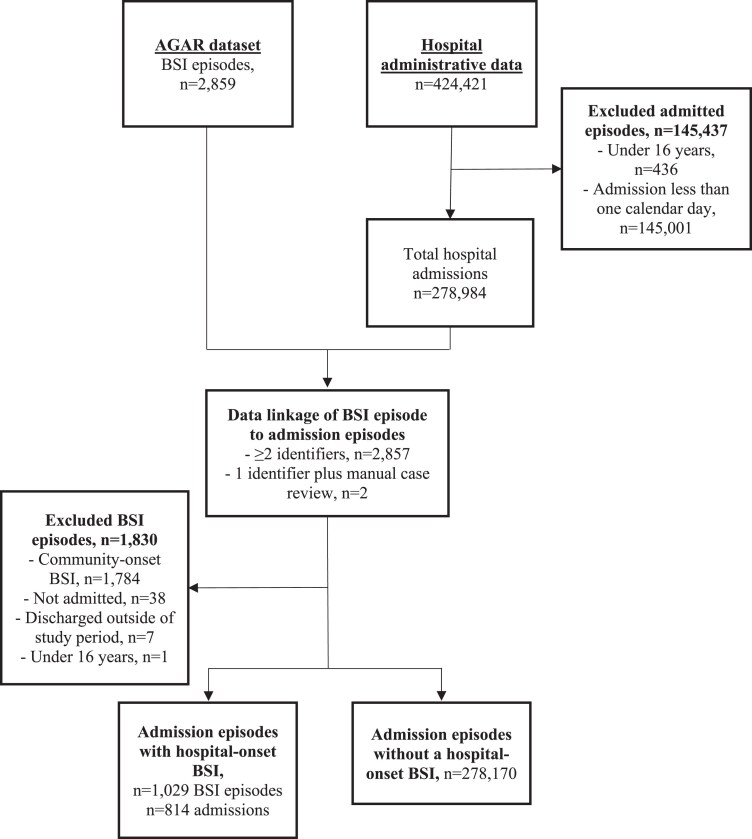
Flowchart of study participants and datasets.

**Figure 2.**
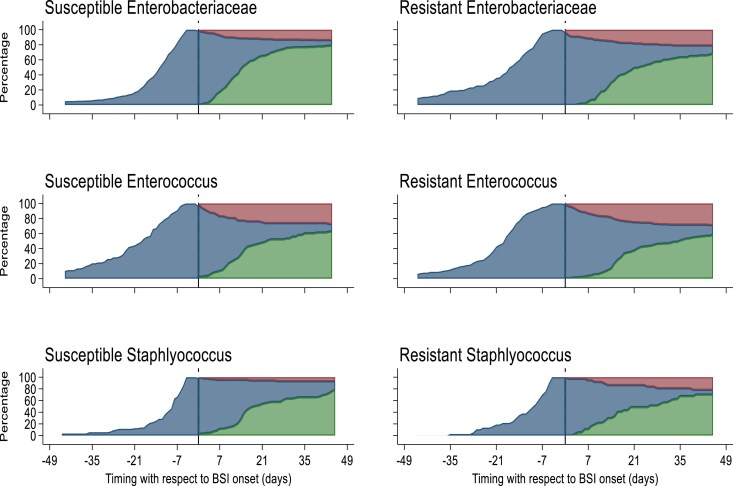
Time to bloodstream infection onset, by antimicrobial susceptibility for each bacterial group. Vertical line = time of hospital-onset bloodstream infection, blue area = patients in hospital, red area = all-cause in-hospital mortality, green area = discharged alive.

The cohort included 278 984 admissions involving 157 728 patients, and 814 (0.3%) admissions had at least one HO-BSI (Figure 1). Patients with an HO-BSI were more frequently male (65.6%) compared with patients without an HO-BSI (54.7%) (Table 1). The median age among HO-BSI episodes was 63 years (IQR: 52–70) compared with admissions without an HO-BSI, 57 years (IQR: 37–73). The median LOS was higher for admissions with an HO-BSI, 31 days (IQR: 21–47), compared with admissions without an HO-BSI, 1 day (IQR: 1–4). In-hospital mortality occurred in 19% (155/814) of admissions with an HO-BSI, compared with 1.5% (4185/278 170) of admissions without an HO-BSI. In-hospital mortality was more frequent for admissions with an antimicrobial-resistant isolate than an antimicrobial-susceptible isolate for all bacterial groups (Table 2). Overall, time from admission to HO-BSI onset was shorter for all antimicrobial-susceptible isolates compared with each bacterial group’s antimicrobial-resistant isolates (Figure 2).

**Table 1.** Patient characteristics for hospital admission episodes, by patient admissions with and without a hospital-onset bloodstream infection (BSI), 2015–2020

Patient characteristic	All admission episodes	Admission episodes without a hospital-onset BSI	Admission episodes with a hospital-onset BSI
	*n* (%) or median [IQR]	*n* (%) or median [IQR]	*n* (%) or median [IQR]
Total hospital admission episodes	278 984	278 170	814
Total unique patients	157 728	157 339	389
Male sex^a^	86 273	86 018 (54.7)	255 (65.6)
Age, y	57 [37, 73]	57 [37, 73]	63 [52, 70]
Age ≥ 65	108 630 (38.9)	108 274 (38.9)	356 (43.7)
Admission source			
Home	252 289 (90.4)	251 647 (90.5)	642 (78.9)
Non-acute ward or facility^b^	1737 (0.6)	1736 (0.6)	1 (0.1)
Acute care	24 958 (8.9)	24 787 (8.9)	171 (21.0)
Emergency admission	218 081 (78.2)	217 634 (78.2)	447 (54.9)
Previous discharge within the past 30 days from Alfred Hospital	39 794 (14.3)	39 696 (14.3)	98 (12.0)
Surgery during admission	153 267 (54.9)	152 653 (54.9)	614 (75.4)
ICU stay during admission	9434 (3.4)	9132 (3.3)	302 (37.1)
Length of stay, d	1 [1, 4]	1 [1, 4]	31 [22, 47]
In-hospital mortality	4340 (1.6)	4185 (1.5)	155 (19.0)
Comorbidities			
Cardiovascular disease	7343 (2.6)	7293 (2.6)	50 (6.1)
Congestive heart failure	11 270 (4.0)	11 139 (4.0)	131 (16.1)
Peripheral vascular disease	3506 (1.3)	3484 (1.3)	22 (2.7)
Cerebrovascular disease	6687 (2.4)	6633 (2.4)	54 (6.6)
Dementia	2756 (1.0)	2753 (1.0)	3 (0.4)
COPD	11 393 (4.1)	11 356 (4.1)	37 (4.5)
Connective tissue disease	1066 (0.4)	1059 (0.4)	7 (0.9)
Peptic ulcer disease	859 (0.3)	845 (0.3)	14 (1.7)
Mild liver disease	10 007 (3.6)	9937 (3.6)	70 (8.6)
Diabetes without end-organ damage	26 120 (9.4)	25 987 (9.3)	133 (16.3)
Diabetes with end-organ damage	24 361 (8.7)	24 212 (8.7)	149 (18.3)
Hemiplegia or paraplegia	2257 (0.8)	2239 (0.8)	18 (2.2)
Renal disease	11 582 (4.2)	11 486 (4.1)	96 (11.8)
Neoplasia	17 734 (6.4)	17 302 (6.2)	432 (53.1)
Metastatic cancer	6351 (2.3)	6310 (2.3)	41 (5.0)
Liver diseases	1366 (0.5)	1328 (0.5)	38 (4.7)
HIV	2424 (0.9)	2413 (0.9)	11 (1.4)

^a^Reported from total unique patients.

^b^Non-acute included transfers from aged care facilities, transition care for older people and residential mental health services.

**Table 2.** Results of proportional hazards models for hospital mortality and discharge alive^a^

Bloodstream infection bacterial group^b^	Admissions with an infection,^c^ *n*	Died, *n*	Incidence per 10 000 days at risk	Unadjusted models^d^		Adjusted models^d^	
				Mortality uHR (95% CI)	Discharge alive uHR (95% CI)	Mortality aHR (95% CI)	Discharge alive aHR (95% CI)
Enterobacterales^e^	403^c^	65 (16.1%)	3.62	2.54 (1.97, 3.28)	0.64 (0.59, 0.68)	2.30 (1.54, 3.42)	0.82 (0.75, 0.91)
Susceptible	299^c^	41 (13.7%)	2.66	2.21 (1.61, 3.03)	0.67 (0.61, 0.72)	1.88 (1.12, 3.15)	0.88 (0.79, 0.97)
Resistant	111^c^	24 (21.6%)	1.01	3.00 (1.98, 4.55)	0.56 (0.50, 0.64)	2.88 (1.78, 4.65)	0.71 (0.59, 0.85)
Enterococci	256^c^	79 (30.9%)	2.34	4.70 (3.72, 5.96)	0.52 (0.47, 0.57)	4.25 (3.24, 5.58)	0.71 (0.63, 0.81)
Susceptible	88^c^	23 (26.2%)	0.83	4.08 (2.68, 6.22)	0.61 (0.51, 0.72)	4.07 (2.65, 6.24)	0.77 (0.62, 0.96)
Resistant	176^c^	57 (32.4%)	1.59	4.57 (3.49, 6.00)	0.49 (0.44, 0.55)	4.03 (2.90, 5.60)	0.69 (0.59, 0.79)
*S. aureus* ^e^	128	17 (13.3%)	1.11	1.55 (0.96, 2.48)	0.49 (0.44, 0.56)	1.70 (0.99, 2.91)	0.55 (0.47, 0.64)
Susceptible	84	9 (10.7%)	0.735	1.18 (0.62, 2.25)^f^	0.48 (0.42, 0.56)	1.50 (0.74, 3.07)	0.51 (0.43, 0.62)
Resistant	37	8 (21.6%)	0.31	2.85 (1.43, 5.69)	0.51 (0.41, 0.63)	3.22 (1.69, 6.14)	0.62 (0.45, 0.85)
Non-fermenting gram-negative bacilli	88	13 (14.8%)	0.80	2.04 (1.16, 3.56)	0.60 (0.52, 0.69)	1.59 (0.83, 3.03)	0.64 (0.52, 0.80)

aHR, adjusted hazard ratio; uHR, unadjusted hazard ratio.

^a^Tests of the proportional-hazards assumptions were *P* > 0.05 or survival lines were roughly parallel, otherwise models were stratified, as specified.

^b^Susceptible and resistant frequencies may not concur with overall total for bacterial group due to admissions with more than one HO-BSI: for admissions with both a resistant and susceptible infection, the resistant infection was used in the resistant model and the susceptible infection in the susceptible model, and only the earlier of the two infections was used in the overall model.

^c^Hospital stays were censored at 45 days.

^d^Reference group is no HO-BSI.

^e^Susceptibility data not available for *n* = 2 Enterobacterales and *n* = 7 *S. aureus* infections.

^f^Stratified by admission type.

### Incidence

There were 1029 unique HO-BSI episodes across 814 admissions. Enterobacterales were the most frequent cause of HO-BSI, with an estimated incidence of 3.62 per 10 000 days at risk, followed by enterococci (2.34 per 10 000 days at risk), *S. aureus* (1.11 per 10 000 days at risk), and NFGNB (0.80 per 10 000 days at risk) (Table 2). HO-BSIs were more frequently antimicrobial-susceptible among Enterobacterales and *S. aureus* but were more frequently antimicrobial-resistant among enterococci (Table 2).

### Attributable mortality

HO-BSIs with all four main bacterial groups were associated with a reduction in hazard of discharge alive compared with patients without HO-BSIs, after adjusting for confounders (Table 2). In addition, HO-BSIs with enterococci and Enterobacterales were associated with an increase in hazard of in-hospital death when compared with admissions without an HO-BSI, after adjusting for confounders. There was no association between *S. aureus* or NFGNB and in-hospital mortality hazard, but the impact of HO-BSI with these pathogens on hazard of discharge alive translated to a higher cumulative risk of death.

The same analysis was performed for each antimicrobial resistance phenotype for *S. aureus*, enterococci and Enterobacterales. For all three bacterial groups, both antimicrobial-susceptible and -resistant HO-BSIs were associated with a reduction in the hazard of discharge alive compared with patients without HO-BSI (Table 2). Except for methicillin-susceptible *S. aureus* (MSSA), all phenotypes were associated with an increase in the hazard of in-hospital death.

### Excess LOS

Patients with an HO-BSI from NFGNB and *S. aureus* bacterial groups had a reduced hazard (daily risk) of the admission ending (all cause end-LOS adjusted HR), corresponding to an increase in LOS. There was no association between HO-BSI caused by Enterobacterales or enterococci and hazard of all-cause end-LOS (Table 3). When stratifying by antimicrobial susceptibility phenotype, none of the bacterial groups except MSSA reduced the hazard of the admission ending compared with patients without HO-BSI.

**Table 3.** Results of proportional hazards analysis for all-cause end-LOS and excess LOS estimates from multistate models^a^

Bloodstream infection bacterial group	All cause end-LOS uHR (95% CI)	All cause end-LOS aHR (95% CI)	Excess LOS, d (95% CI)
Enterobacterales	0.72 (0.68, 0.77)	0.92 (0.84, 1.01)	4.54 (4.21, 4.86)
Susceptible	0.74 (0.68, 0.80)	0.95 (0.86, 1.06)	4.14 (3.82, 4.46)
Resistant	0.68 (0.61, 0.77)	0.84 (0.71, 1.01)	5.67 (4.88, 6.47)
Enterococci	0.72 (0.66, 0.78)	0.97 (0.87, 1.08)	4.41 (4.05, 4.78)
Susceptible	0.78 (0.68, 0.90)	0.98 (0.80, 1.20)	3.12 (2.61, 3.63)
Resistant	0.69 (0.63, 0.75)	0.95 (0.84, 1.08)	4.94 (4.47, 5.40)
*S. aureus*	0.54 (0.49, 0.60)	0.60 (0.52, 0.70)	8.67 (8.02, 9,32)
Susceptible	0.52 (0.45, 0.59)	0.55 (0.46, 0.66)	9.79 (9.05, 10.5)
Resistant	0.62 (0.52, 0.74)	0.75 (0.55, 1.02)	6.30 (5.34, 7.25)
Non-fermenting gram-negative bacilli	0.67 (0.59, 0.77)	0.71 (0.58, 0.87)	5.55 (4.76, 6.35)

aHR, adjusted hazard ratio; LOS, length of stay; uHR, unadjusted hazard ratio.

^a^Tests of the proportional-hazards assumptions were *P* > 0.05 or survival lines were roughly parallel.

Of the four main bacterial groups, HO-BSI with *S. aureus* had the greatest attributable impact on excess LOS (9 days for *S. aureus* and 4–5 days for other bacterial groups, compared with admissions without HO-BSI). The estimated excess LOS due to HO-BSI with antimicrobial-resistant isolates (which represent the estimate difference in LOS compared with the expected LOS had the resistant HO-BSI not occurred) ranged between 5 and 6 days (Table 3). Estimated excess LOS was slightly lower for admissions with antimicrobial-susceptible isolates for Enterobacterales and enterococci BSIs, but higher for admissions with antimicrobial-susceptible *S. aureus* BSIs. Excess LOS attributable to susceptible *S. aureus* may appear greater than for resistant *S. aureus* because the latter has a higher in-hospital mortality rate: the median time from BSI onset to death (among those who died) was shorter than time from BSI onset to discharge alive (among those who survived) (Table S1; available as *Supplementary data* at *JAC-AMR* Online).

## Discussion

We describe the burden of HO-BSI caused by four priority pathogen groups—overall and stratified by major resistance phenotype—in an Australian healthcare facility using linked surveillance data. Linkage allowed us to estimate both the incidence of HO-BSI (per 1000 patient-days at risk) and the outcomes: attributable mortality and excess LOS. As competing events, the impacts of HO-BSIs on the hazard (i.e. daily risk) of in-hospital mortality and discharge alive need to be interpreted together to appreciate their cumulative impact on attributable mortality. All HO-BSI bacterial groups, and both antimicrobial-susceptible and -resistant isolates within each group, reduced the daily risk (hazard) of discharge alive, compared with admissions without HO-BSI. This alone will tend to result in an increased cumulative risk of in-hospital death by extending the time at risk (duration of admission). In addition, infection with all HO-BSI groups, and both antimicrobial-susceptible and -resistant subgroups, increased the daily risk (hazard) of death, except for *S. aureus*, MSSA and NFGNB, compared with admissions without HO-BSI. In those three groups, we estimated a clinically significant increase in hazard of death. Although we did not directly compare patients with antimicrobial-resistant and -susceptible infections, infections with resistant isolates appear to have a substantially greater impact on mortality compared with susceptible isolates for Enterobacterales and *S. aureus*. The risk of mortality was highest for enterococci HO-BSI but was similar for both vancomycin-susceptible and -resistant isolates, when compared with those without an HO-BSI.

Our results can be compared with previous studies that applied similar methods in different settings, although differences in methodology do complicate this. Lee *et al.*^6^ linked microbiology and administrative data to assess the impact of healthcare-associated BSI on mortality and hospital LOS in Queensland, Australia, but direct comparison with our study is difficult as their results were reported as odds ratios (logistic regression models) and somewhat different bacterial groups were assessed. In our study, when analysed by antimicrobial susceptibility, the adjusted hazard of death was 3.22 for patients with methicillin-resistant *S. aureus* (MRSA), which is higher than in a study involving 10 European hospitals (2.42), and lower than a study involving 9 Australian hospitals (4.6). The adjusted hazard of death for patients with MSSA was lower (1.50) compared with both the other studies (1.8 and 3.4, respectively).^10,16^ The hazard of death for third-generation cephalosporin–susceptible and –resistant Enterobacterales was higher in our study compared with other studies, which have reported considerable heterogeneity in results.^9,10,17,18^ In our study, there was a higher hazard of death for both susceptible and resistant Enterobacterales, when each were compared with admissions without HO-BSI. Similarly, in European hospitals and low-income and middle-income country sites, there was often minimal impact of cephalosporin resistance in Enterobacterales on mortality when compared with susceptible BSI with the equivalent species.^9,10,17,18^

Excess LOS due to HO-BSI was higher for antimicrobial-resistant Enterobacterales and enterococci, than the respective bacterial group’s antimicrobial-susceptible isolates, when each was compared with admissions without HO-BSI. However, overall, excess LOS was highest for MSSA. The overall variation reflects existing literature that reports little or no evidence of variation in LOS by antimicrobial susceptibility across groups of causative organisms.^5,9,10,16,17^ Our findings are also consistent with AGAR reports that the proportion of patients with an LOS of more than 30 days was higher for patients with MSSA BSI compared with MRSA BSI.^16^ The difference between resistant and susceptible phenotypes in excess LOS in our study for *S. aureus* is likely driven by the higher mortality hazard for the resistant phenotype, which reduces the time from infection to death. This highlights that LOS may be a perverse marker of morbidity where mortality is high and the need to consider mortality when using LOS for cost calculation as a marker of healthcare system burden.^6,19^ Interestingly, Lee *et al*.^6^ estimated that MRSA had a larger impact on excess LOS than MSSA, whereas we found the reverse despite using the same method. This difference may be related to the greater difference in all-cause mortality among patients with MRSA and MSSA BSI in our data (21.6% and 10.7%, respectively) compared with Lee’s study (12.9% and 13.4%, respectively) as high mortality shortens LOS, further highlighting that LOS may be a perverse marker of morbidity.

We demonstrated a successful proof-of-concept, that the nationally implemented AGAR surveillance system can be used to generate estimates of the burden of BSI through linkage to hospital administrative data. Through this linkage, we estimated attributable mortality, rather than crude mortality, which is a valuable addition to the current AGAR surveillance. AGAR routinely reports the proportion of patients with 30 day all-cause mortality from date of blood culture collection, although there is no post-discharge follow-up for patients who are discharged before 30 days, and no adjustment for confounders. Consequently, the metric cannot be considered to reflect the mortality attributable to BSI. In contrast, here we quantified the mortality attributable to HO-BSI by estimating the adjusted hazard of in-hospital mortality and discharge alive, to account for confounding, time-dependent bias and competing events. Our approach could be replicated across multiple AGAR sites to provide additional insights across other geographic areas. It could also be performed for community-onset BSIs, which were excluded from our analysis, and have distinct microbial profiles and antimicrobial resistance patterns, and on the economic impact, which has only been quantified through simulation models in Australia.^20^

Our study has some limitations. First, our study was a retrospective single-site study, which restricts the ability to extrapolate results to different regions and hospital types in Australia. Second, although we adjusted for several important confounders, our retrospective analysis did not adjust for clinical and therapeutic variables and may be vulnerable to residual confounding. Third, our study was limited to the high-priority bacterial groups included in AGAR surveillance, which does not include some important causes of BSI, such as *Candida* species, and therefore underestimates total BSI burden. Fourth, we were limited in our ability to complete the analysis for other NFGNB by antimicrobial susceptibility due to small sample size. Fifth, our analysis focused on hospital-onset infections, which can be derived from hospital administrative data, rather than applying resource-intensive manual definitions of HAIs. This approach may be vulnerable to misclassification bias, but we note that ‘hospital-onset bloodstream infection’ is now a widely applied infection prevention metric.^21,22^ Finally, we did not directly estimate the impact of resistant infections compared with susceptible infections, which would be particularly relevant if we assume that resistant infections replace susceptible infections. Instead, with our comparator of uninfected patients, we assumed that resistant infections contribute to the total burden of infections.^23^ In a framework to guide comparator selection when estimating the burden of antimicrobial resistance, an intervention-based causal approach emphasizes the need to understand the trajectory of antimicrobial usage to facilitate the most relevant comparator; however, these data were not available in our study.^23^ We selected our approach in order to quantify the burden of disease that could be averted with infection prevention strategies that prevent both susceptible and resistant HO-BSIs and contributed to the scant literature that uses uninfected patients as the comparator.^23,24^

HO-BSIs have a substantial impact on patient outcomes, with an increased risk of death and excess LOS for both antimicrobial-resistant isolates and antimicrobial-susceptible isolates when compared with patients without HO-BSI. This approach of linking AGAR’s laboratory-based BSI surveillance with administrative datasets could be scaled-up at all sites to provide national information about the attributable burden of HO-BSI.

## Supplementary Material

dlaf183_Supplementary_Data

## Data Availability

The data that support the findings of this study are available from the corresponding author, S.J.C., upon reasonable request, subject to approval by the relevant institutional review boards.

## Supplementary data

Table S1 is available as Supplementary data at *JAC-AMR* Online.
