# Supplementary material for: Mortality and length of stay associated with antimicrobial-susceptible and -resistant hospital-onset bloodstream infections at a tertiary referral hospital in Melbourne, Australia
Source: JAC Antimicrob Resist. 2025 Oct 21;7(5):dlaf183. doi: 10.1093/jacamr/dlaf183 (PMC12539618; doi:10.1093/jacamr/dlaf183)
Supplement: dlaf183_Supplementary_Data [file dlaf183_supplementary_data.docx]

**Supplementary Material 1. Length of stay, time from hospital admission to hospital-onset bloodstream infection and time from hospital-onset bloodstream infection to death or discharge, by antimicrobial-susceptible and resistant isolates of bacterial groups**

|  | **Admissions with an**  **infection, n** | **Length of stay,**  **median (IQR)** | **Time to infection, median (IQR)** | **Time to death or discharge, / median (IQR)** |  |
| --- | --- | --- | --- | --- | --- |
| **Enterobacterales** | | | | |  |
| Overall | 403 |  |  |  |  |
| Alive | 338 | 29 (21, 39) | 13 (8, 18) | 14 (9, 20) |  |
| Died | 65 | 23 (16, 37) | 13 (9, 18) | 7 (1, 13) |  |
| Resistant | 111 |  |  |  |  |
| Alive | 87 | 34 (26, 45) | 15 (9, 21) | 15 (10, 23) |  |
| Died | 24 | 33 (18, 43) | 18 (11, 32) | 6 (1, 16) |  |
| Susceptible | 299 |  |  |  |  |
| Alive | 258 | 27 (20, 37) | 12 (8, 17) | 14 (9, 20) |  |
| Died | 41 | 19 (15, 31) | 10 (7, 17) | 7 (2, 11) |  |
| **Enterococci** | | | | |  |
| Overall | 256 |  |  |  |  |
| Alive | 177 | 37 (30, 45) | 18 (13, 23) | 16 (12, 22) |  |
| Died | 79 | 28 (20, 44) | 16 (11, 25) | 8 (3, 15) |  |
| Resistant | 176 |  |  |  |  |
| Alive | 119 | 38 (31, 45) | 18 (14, 23) | 17 (13, 22) |  |
| Died | 57 | 30 (21, 45) | 17 (13, 24) | 9 (4, 17) |  |
| Susceptible | 88 |  |  |  |  |
| Alive | 65 | 35 (25, 45) | 17 (13, 24) | 14 (9, 20) |  |
| Died | 23 | 28 (14, 43) | 15 (8, 30) | 7 (2, 13) |  |
| ***Staphylococcus aureus*** | | | | | |
| Overall | | 128 |  |  |  |
| Alive | | 111 | 29 (21, 45) | 8 (6, 14) | 16 (12, 28) |
| Died | | 17 | 21 (12, 45) | 7 (5, 14) | 13 (6, 26) |
| Resistant | | 37 |  |  |  |
| Alive | | 29 | 31 (20, 40) | 10 (6, 15) | 16 (10, 32) |
| Died | | 8 | 18.5 (14, 43.5) | 7.5 (4.5, 20.5) | 12.5 (8, 26.5) |
| Susceptible | | 84 |  |  |  |
| Alive | | 75 | 29 (22, 45) | 8 (6, 13) | 16 (13, 29) |
| Died | | 9 | 30 (10, 45) | 6 (5, 13) | 17 (4, 29) |
